# Mucosal Associated Invariant T Cells Were Activated and Polarized Toward Th17 in Chronic Obstructive Pulmonary Disease

**DOI:** 10.3389/fimmu.2021.640455

**Published:** 2021-03-31

**Authors:** Wenjia Qiu, Ning Kang, Yanxu Wu, Yongjun Cai, Li Xiao, Haiyan Ge, Huili Zhu

**Affiliations:** ^1^ Department of Respiratory Medicine, The Affiliated Huadong Hospital of Fudan University, Shanghai, China; ^2^ Department of Thoracic Surgery, The Affiliated Huadong Hospital of Fudan University, Shanghai, China; ^3^ Department of Pathology, The Affiliated Huadong Hospital of Fudan University, Shanghai, China

**Keywords:** mucosal associated invariant T cell, chronic obstructive pulmonary disease, interleukin-17, activation, cytokine

## Abstract

Chronic obstructive pulmonary disease (COPD) is a chronic inflammatory disease characterized by airway limitation accompanied with infiltration of inflammatory cells. Mucosal associated invariant T (MAIT) cells can recognize bacteria and play an important role in controlling host immune responses by producing cytokines. In this study, we characterized the function and the ability of MAIT cells to secrete cytokines measured by flow cytometry. In COPD patients, MAIT cells have the ability to produce more IL-17 and less IFN-γ compared to healthy individuals. We found that HLA-DR expression levels reflected the degree of inflammation and the proportion of IL-17 was significantly correlated with lung function in peripheral blood. In addition, we found that MAIT cells were highly expressed in the lung, and the increased expression of CXCR2, CXCL1 indicated that MAIT cells had the potential to migrate to inflammatory tissues. This evidence implies that MAIT cells may play a potential role in COPD immunopathology.

## Introduction

Chronic obstructive pulmonary disease (COPD) is the most common cause of chronic respiratory disease attributable to deaths (1, 2). Chronic inflammation of COPD is mediated by innate immunity (neutrophils) and adaptive immunity (CD4+ T cells), especially in small airways, the degree of inflammation correlates with the severity of the disease classified by the Global Initiative for Chronic Obstructive Lung Disease (GOLD) (3–6).However, recent evidences illustrate that innate immune-like cells are equivalently important involved in the pathogenesis of COPD. ILC and unconventional T cells(iNKT, Mucosa-associated invariant T cells, and γδ-T cells) make up only a small part of the total immune cells, but they have been found to maintain pulmonary homeostasis and protect the integrity of lung epithelium (7–11).

MAIT cells are the recently discovered innate-like lymphocytes that manifest restricted variety T cell receptor (TCR) and exhibit the semi-invariant TCRVα7.2-Jα33 in human (12). MAIT cells can recognize bacteria and fungi derived from riboflavin metabolites through APC-mediated MHC I-related molecules (MR1) (13). It has been reported that MAIT cells have the ability to produce diverse cytokines in response to IL-4, IL-5, IL-12, IL-18 and release cytotoxic molecules (granzyme and perforin) to kill infected host cells indirectly (14–17). MAIT cells are abundant in mucosal tissues (18) but decreased in blood and play a critical role in infectious diseases and metabolic disorders.

In patients infected with bacteria and virus, the frequency of MAIT cells has been shown to be reduced in the blood (14, 19–21). In intestinal (22) and liver (23, 24) studies, MAIT cells are activated to migrate to inflamed tissues and modulates immune responses in mucous tissues. Previous studies (10, 25, 26) have noted MAIT cells are reduced in peripheral blood in COPD patients, nevertheless, the role of MAIT cells in COPD remains unclear and yet to be unraveled.

Due to the unique retinoic acid-related orphan receptor (RORγt) and CD161^hi^ surface molecules which usually served as signature factor for Th17 cells (18), we hypothesized migration from peripheral blood to inflammatory tissues was due to activation of MAIT cells. The cytokines produced by the activated MAIT cells in COPD patients may promote the immune system to skew toward Th17. In this study, we attempted to define phenotype, clinical relevance, and functions of MAIT cells in COPD patients. And we try to determine the mechanism responsible for MAIT cell reduction in peripheral blood.

## Materials

### Subjects and Ethical Statement

COPD patients and healthy individuals were recruited in Huadong Hospital, Fudan University. The diagnosis of COPD was based on Global Initiative for Chronic Obstructive Lung Disease (GOLD) (27). All subjects had no history of autoimmune diseases, recent surgery, malignancies, or use of immunosuppressive drugs. The clinical characteristics of the subjects were described in **Table 1**. In order to verify the migration of MAIT cells, we collected pulmonary parenchyma from additional COPD patients undergoing surgery for small nodules. Control tissues were selected who had lung lobectomy because of small nodules or pneumothorax, and these patients had no problems with lung function. We confirmed that the harvested tissue did not contain tumor lesions. The studies involving human participants were reviewed and approved by the ethics committee of Huadong Hospital. And informed consents were obtained from all subjects with the Declaration of Helsinki.

**Table 1:** Clinical and laboratory characteristics of patients and healthy controls.

	Health Individuals (n = 50)	COPD Subjects (n = 75)
Total, n	50	75
Sex, Male/Female, n	30/20	68/7
Age (mean ± SD), years	67.82 ± 11.37	71.25 ± 9.63
BMI, kg/m^2^	23.89 ± 3.14	23.65 ± 3.38
Current-smokers/Former-smokers/Non-smokers	28/10/12	21/37/17
Smoking (pack-years)	23.33 ± 39.83	34.76 ± 35.47
**Clinical variables (mean ± SD)**		
Pre FEV_1_ predicted (%)	110.29 ± 19.75	51.28 ± 17.76
Post FEV_1_ predicted (%)	NA	54.43 ± 18.00
FVC predicted (%)	94.87 ± 15.58	69.03 ± 18.52
FEV_1_/FVC (%)	92.16 ± 10.2	60.83 ± 9.31
DLCO (%)	76.0 ± 21.26	50.85 ± 22.74
TLC (%)	123.98 ± 17.31	149.54 ± 18.11
FEF50(%)	117.71 ± 38.77	25.66 ± 15.12
Duration of COPD	NA	8.06 ± 8.30
GOLD stage,1/2/3/4	NA	11/22/32/8
Frequent exacerbator phenotype, >2/yr	NA	0.73 ± 0.90
mMRC dyspnea Score	NA	2.00 ± 1.10
CAT Score	NA	15.37 ± 7.23
SGRQ Score	NA	24.68 ± 13.57
Inhaled steroids (%)	NA	34 (45.3%)
**Laboratory variables (mean ± SD)**		
Leukocytes (*10^9^/L)	5.99 ± 1.18	6.92 ± 1.92
Neutrophils (cells/μl)	2.78 ± 1.64	4.61 ± 1.63
Lymphocytes (cells/μl)	1.85 ± 0.60	1.57 ± 0.68
Eosinophils (cells/μl)	0.14 ± 0.10	0.20 ± 0.16
Monocyte (cells/μl)	0.37 ± 0.12	0.47 ± 0.17
Hemoglobin (g/dl)	129.06 ± 18.03	139.27 ± 20.26
RDW (%)	12.96 ± 1.16	13.25 ± 1.13
Platelets (10^3^ cells/μl)	216.91 ± 52.77	199.76 ± 62.51
CRP level (mg/dl)	6.70 ± 8.27	8.7 ± 12.37
ESR (mm/hour)	16.17 ± 20.22	27 ± 48.43
**Co-morbid conditions, n (%)**		
Hypertension	18 (36%)	24 (32%)
Cerebrovascular disease ddisediseasecorticosteroids, Y/N	2 (4%)	4(5.3%)
Diabetes mellitus	5 (10%)	6(8%)
Allergic rhinitis	2 (4%)	8(10.7%)

SD, standard deviation; COPD, chronic obstructive pulmonary disease; BMI, body mass index; FEV1, forced expiratory volume in 1 s; FVC, forced vital capacity; FEF50, the forced expiratory flows between 50% of the FVC; GOLD, Global Initiative for Chronic Obstructive Lung Disease; mMRC, modified Medical Research Council; CAT, COPD assessment test; SGRQ, St George’s Respiratory Questionnaire; NA, not applicable; RDW, Red blood cell distribution width; CRP, C-reactive protein; ESR, erythrocyte sedimentation rate; ICS, inhaled corticosteroids.

### Flow Cytometric Analysis and Surface Marker

Peripheral venous blood samples were collected into the EDTA-anticoagulant tubes within 4 h. Both bloods lysed by BD Lysing Buffer (BD Biosciences) and PBMC were stained for surface antigen for 30 min at room temperature. Dead cells were stained for exclusion by using Zombie AquaTM Dye (Biolegend) for 15 min. MAIT cells were identified as CD3^+^ TCRγδ^−^ CD161^high^TCRVα7.2^+^cells (18, 22, 28). And MR1 tetramer was defined to specifically detect MAIT cells (14). The following monoclones were used: CD3 (OKT3), CD4 (A161A1), CD8(SK1), TCRγδ (B1), CD161 (HP3G10), TCRVα7.2 (3C10), CD127 (A019D5), CCR5 (HEK/1/85a), HLA-DR (L243) from Biolegend. All flow cytometry operations were performed on FACS Arial II (Beckton Dickinson) and data was analyzed by flowjo V10.

### Isolation of Peripheral Blood Mononuclear Cells and Cytokine Stimulation

EDTA-blood was diluted 1:1 with phosphate buffered saline (PBS, Hyclone) and purified by density-gradient centrifugation with Lymphoprep™ (AXIS-SHIELD). PBMCs were cultured in RPMI 1640 medium (Hyclone) containing 10% fetal bovine serum (FBS, Gibco) with 100 units/ml of penicillin (Gibco) at a concentration of 1 × 10^6^ cells/ml in a 37°C cell culture incubator with 5% CO2.Expression levels of cytokines were detected by intracellular cytokines protocol as previously described (29, 30). Freshly drawn peripheral blood samples were stimulated for 5 h with 250 ng/ml PMA (Sigma) and 1 μg/ml ionomycin (PeproTech). After 1 h, 1 μl brefeldin A (Golgi Plug; BioLegend) was added. For intracellular staining, the fixation and Intracellular Staining Perm Wash Buffer was utilized according to the manufacturer’s protocol (Biolegend). Cells were stained using mAbs specific for IFN-γ (4S.B3), IL-17(BL168), and IL-22 (2G12A41) (Biolegend).

### Stimulation of PBMCs With IL-7

PBMCs were cultured at a concentration of 1 × 10^6^ cells/ml in RPMI 1640 medium (Hyclone) and incubated with 10 ng/ml IL-7 (PeproTech) at 37°C for 30 min to quantify STAT-3 phosphorylation(pY705) or analyze surface molecules and RORγt(AFKJS-9) after 24 h. Clones of antigen of MAIT cells were as described above.

### Isolation of Human Lung Primary Cells

As protocol described (31, 32), human lung tissues were washed with HBSS(Hyclone) with 2% FBS, cut into small pieces and digested for 30 min with DNA enzyme and collagenase at 37°C with agitation. Fill the tube of small pieces of lung tissue with HBSS, centrifuge at 500 g for 5 min, using 100 μm filter to obtain cell suspension and repeated twice. Red blood cells were lysed using 5 ml of lysis buffer (Biolegend) for 5 min and washed with HBSS/2% FBS. Cell lysate was resuspended in RPMI 1640 medium. The subsequent stimulation steps are as described reference.

### Quantitative Real-Time PCR Analysis

Total RNA was isolated with Total RNA Extraction Reagent (EZBioscience) following the instructions of manufacturer. Quantitative real-time PCR using SYBR Green PCR mixture (TAKARA) was to quantify the level of MR1, Vα7.2, CCL20, CXCL1, CXCR2, CCL2 with the following conditions 95°C for 30 s, 40 cycles at 95°C for 10 s, and 60°C for 30 s. All experiments were repeated at least third independently to ensure the reproducibility.

### Immunofluorescence

The fresh tissues were sliced into 4 μm section for immunofluorescence (LEICA CM1950). Briefly, the sections were washed with phosphate buffered saline (PBS, Hyclone) at room temperature (China), then were blocked with 10% goat serum at room temperature for 60 min and incubated with CD3 (abcam) and purified Vα7.2(3C10, Biolegend) mixture overnight at 4°C. Slices were incubated with fluorescent secondary antibody mixture for 1 h followed by PBS washing. DAPI (Beyotime) was used for nuclear staining for 5 min. After washing, the sections were covered with Antifade Mounting Medium (Beyotime) and cover glass. Confocal laser scanning microscope (LEICA TCS SP8) was used for detection.

### Immunohistochemistry

All sections were washed with PBS at room temperature. The endogenous peroxidase activity was deactivated with 3% hydrogen peroxide for 20 min. After being blocked with the goat serum for 10 min, the sections were incubated with TCRvα7.2(Biolegend) antibody overnight at 4°C followed by secondary antibody for 1 h at room temperature. The immunostaining was performed by hematoxylin counterstain.

### Statistical Analyses

Statistical analysis was performed by SPSS version 22.0 software and GraphPad Prism version 8.3.0. Mann–Whitney U-test and paired t test were used to analyze differences in continuous variables. Correlations were determined by Spearman’s correlation coefficient. Linear regression analysis was used to test associations between MAIT cell levels and clinical or laboratory parameters. All comparisons of MAIT cells were performed by Kruskal-Wallis test using Dunn’s and Bonferroni for multiple comparisons. Statistical significance was considered when *P* value <0.05 (^*^ *P* < 0.05, ^**^ *P* < 0.01, ^***^ *P* < 0.001).

## Result

### MAIT Cells Among CD3^+^ T Cells Are Decreased in Peripheral Blood of COPD Patients

**Figure 1:**
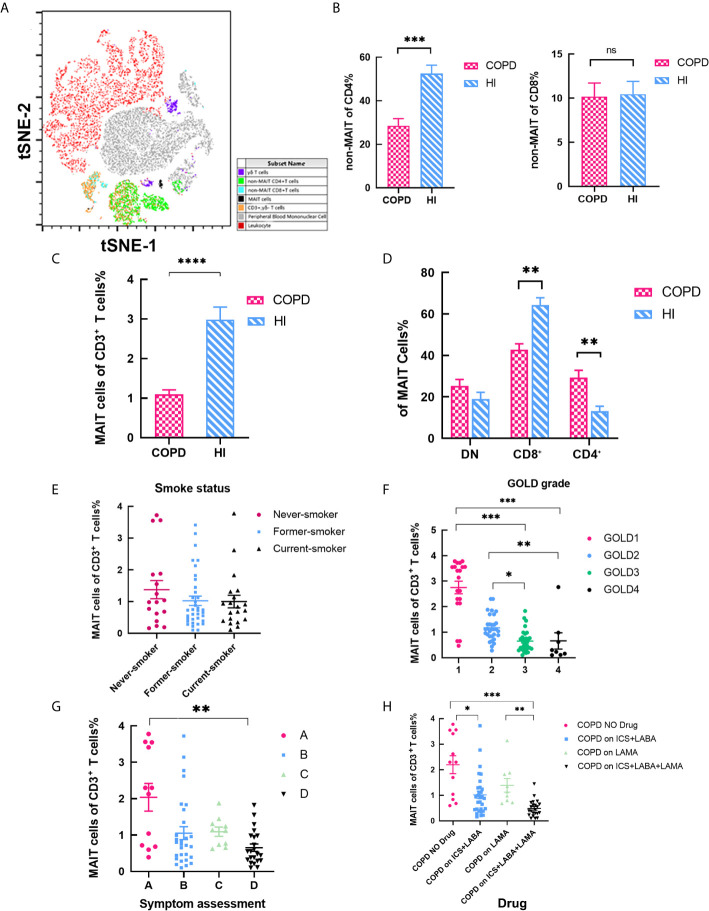
MAIT cells among CD3^+^ T cells are altered in COPD Patient. **(A)** tSNE analysis of flow cytometry scatter plots from COPD and health individuals. CD3^+^ TCRγδ^−^CD161^high^TCR Vα7.2^+^ cells were confirmed as MAIT cells. **(B)** Ratio of non-MAIT CD4 and CD8 T cells in peripheral blood. **(C)** Statistical analysis of circulating MAIT cell frequency in HI (n = 50) and patients with COPD (n = 75). **(D)** Percentages of subset among MAIT cells in peripheral blood. **(E)** Percentage of MAIT cells in peripheral blood according to smoking status. COPD patients with severe COPD grades **(F)** and higher symptom evaluation **(G)** had lower proportion of MAIT cell. **(H)** Proportion of MAIT cells in different drug using. Data were analyzed with Mann–Whitney U-tests and Kruskal-Wallis test with Dunn’s correction for multiple comparisons. Horizontal lines are mean ± SEM values. *P < 0.05, **P < 0.01 ***P < 0.001. HI, health individual; MAIT, mucosal-associated invariant T; DN, double negative; ICS, inhaled corticosteroids; LABA, long-acting beta agonist; LAMA, long-acting muscarinic antagonist. ns, non-significant.

The baseline characteristics of the 75 patients and 50 age-matched health individuals are summarized in **Table 1**. As mentioned earlier, MAIT cells were defined as CD3^+^ TCRγδ^−^CD161^high^ Vα7.2^+^ T cells (**Figure 1A**) (Gating strategy in **Supplemental Figure 1**). The proportion of non-MAIT CD4 T cells was significantly reduced in COPD patients and CD8 T cells was no difference (mean 28.57 *vs.* 52.54%, *P* < 0.001; mean 10.18 *vs.*10.46, respectively; **Figure 1B**). Percentage of MAIT cells was significantly lower in COPD patients compared to HI (mean 1.10 *vs.* 2.99%, *P* < 0.001; **Figure 1C**). We observed biased decreased frequencies of CD8^+^ subpopulations in COPD patients (mean 42.77 *vs.* 64.36%, *P* < 0.01) consistent with previous results (25, 33), since CD4^+^ subset (mean 29.30 *vs.*13.12%) increased, and DN subset (mean 25.35 *vs.*19.06%) proportion was no difference between patients and healthy individuals (**Figure 1D**). In addition, we investigated whether circulating MAIT cells frequency was associated with clinical parameters in COPD patients. Subjects were subdivided into the current-smoker, former-smoker, and never-smoker subgroups according to smoking status. However, no significant differences in circulating percentages of MAIT cells were observed among COPD groups (mean 1.375 *vs.* 1.025 *vs.* 1.00%; **Figure 1E**). According to the percentage of FEV1 to the predicted, the GOLD classification reflects the patient’s lung function status (27), we suggest as the GOLD grade increases, the proportion of cells gradually decreases. This situation also occurs in the symptom scores (mean 2.73 *vs.* 1.17 *vs.* 0.65 *vs.* 0.66%; mean 2.04 *vs.* 1.05 *vs.* 1.09 *vs.* 0.65% respectively; **Figures 1F–G**). The lower the cell frequency, the higher number of acute attacks and hospitalizations. Our data shows corticosteroid could significantly decrease the frequency of MAIT cells. However, LAMA-use alone may not cause reduction of MAIT cells (**Figure 1H**).

### MAIT Cells Were Activated in Peripheral Blood in COPD Patients

**Figure 2:**
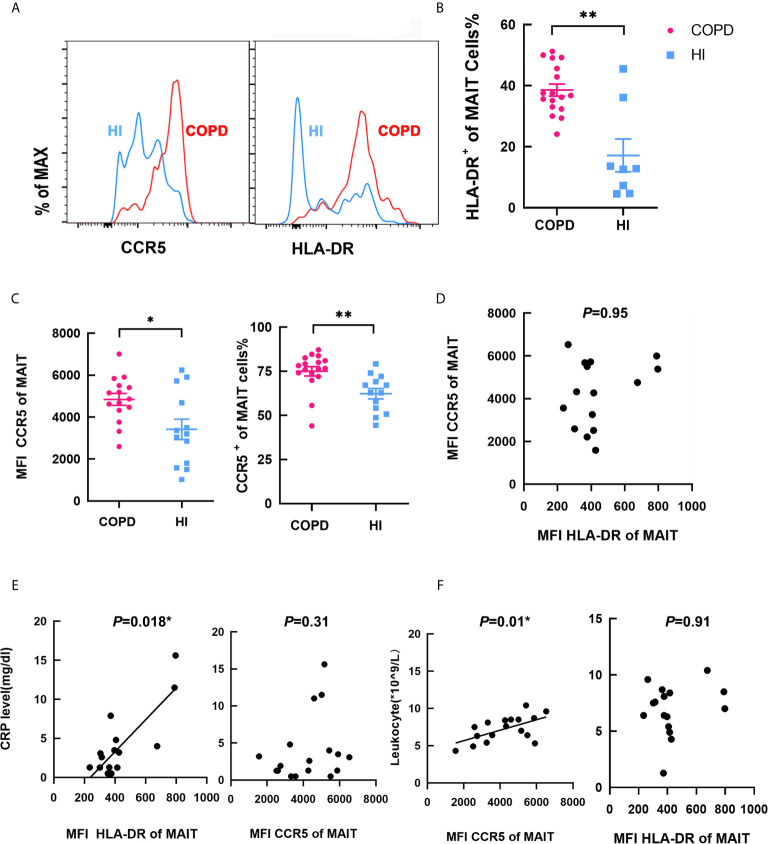
Phenotype of circulating MAIT cells in COPD patients. **(A)** Representative staining in COPD patients and HI. **(B)** Proportion of HLA-DR^+^ on MAIT cells in HI and COPD patients. **(C)** Higher MFI and frequency of CCR5^+^ MAIT cells in COPD patients. **(D)** MFI of HLA-DR was no correlation with MFI of CCR5. **(E)** MFI of HLA-DR was significantly correlated with CRP level (mg/dl) (r = 0.58, *P* = 0.018), whereas MFI CCR5 was not correlated with CRP level (n = 25). **(F)** MFI of CCR5 was significantly correlated with leukocyte (*10^9^/L, r = 0.59, *P* = 0.01) while MFI HLA-DR was not related (n = 25). Symbols represent individual subjects and data was analyzed with Mann–Whitney U-test. *P < 0.05, **P < 0.01. IFN, interferon; MAIT, mucosal-associated invariant T; IL, interleukin; HI, healthy individual; HLA-DR, human leukocyte antigen DR; MFI, mean fluorescence intensity; CCR5, C-C chemokine receptor type 5.

Then, we investigated the function of circulating MAIT cells from HI and patients. The frequency of MAIT cells expressing HLA-DR activation marker were higher in COPD patients compared to HI (mean 38.56 *vs.* 17.14%, *P* < 0.01; **Figures 2A, B**). We found that COPD patients and individuals had high expression of CCR5, and COPD group was slightly higher than that of the health group both in MFI and proportion (**Figure 2C**), indicating their strong ability to migrate to tissues. Our results showed that MFI HLADR was independent of CCR5 expression (**Figure 2D**). CRP level was significantly correlated with MFI of HLA-DR (r = 0.58, *P* = 0.018, **Figure 2E**), but not with CCR5, which was significantly correlated with leucocyte levels (r = 0.59, *P* = 0.01, **Figure 2F**). Notably, consider the relationship between CRP and disease severity, we compare HLA-DR and CCR5 expression relate to patient GOLD grades. The MFI of HLA-DR was decreased, though not statistic significant. However, the MFI of CCR5 was increased in GOLD3 and GOLD4 patients compared to GOLD2 (**Figure S2**).

### IL-17^+^ MAIT Cells increased in Peripheral Blood of COPD Patients

**Figure 3:**
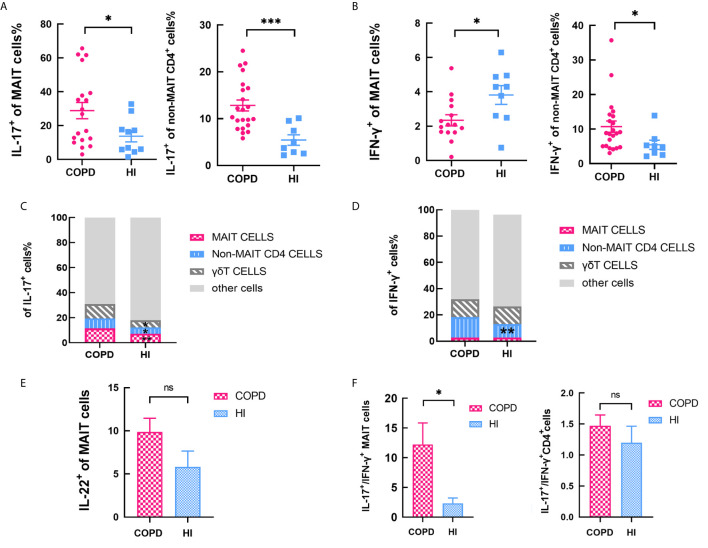
Cytokines of MAIT cells in peripheral blood. **(A)** Percentage IL-17^+^ of MAIT cells increased both in MAIT cells and non-MAIT CD4^+^ T cells with PMA-ionomycin stimulation. **(B)** Percentage of IFN-γ^+^ decreased in MAIT cells while increased in non-MAIT CD4^+^ T cells compared with health. **(C)** Data showing subset of IL-17^+^ cells in the indicated subsets from COPD patients and health individuals. **(D)** Data showing frequency of IFN-γ^+^ cells in the indicated subsets from COPD patients and health individuals. **(E)** Percentage of IL-22^+^ of MAIT cells in circulating MAIT cells from HI and COPD patients with PMA-ionomycin stimulation. **(F)** The ratio of IL-17^+^/IFN-γ^+^ in MAIT cells and non-MAIT CD4^+^ T cells. Data were analyzed with Mann–Whitney U-tests. Horizontal lines are mean ± SEM values. ns, non-significant; *P < 0.05, **P < 0.01, ***P<0.001.

After PMA-ionomycin stimulation, the frequency of circulating IL-17^+^ MAIT cells were significantly higher in COPD patients, and same situation occurs in non-MAIT CD4^+^ T cells (**Figure 3A**). IFN-γ^+^ of MAIT cells was decreased while non-MAIT CD4+ T cells expressed a higher proportion (**Figure 3B**). In the peripheral blood of COPD patients, though the percentage of Th17 cells was more abundant than MAIT cells, IL-17 was produced at significantly increased levels by MAIT cells; 11.5% of MAIT cells and 7% of conventional non-MAIT CD4+ T cells produced IL-17A. In contrast, in the blood of health individuals, 7.6% of MAIT cells and 4.6% of conventional T cells expressed IL-17. We assessed compared with HI, the proportion of cells that produce IL-17 was increased including MAIT cells, γδT cells, non-MAIT cells in COPD patients (**Figure 3C**). Of note, proportion of non-MAIT CD4 cells of IFN-γ^+^ was increased in COPD patients while other groups are comparable (**Figure 3D**). In addition, IL-22^+^ of MAIT cells was no different between COPD patients and healthy individuals (**Figure 3E**). The ratio of peripheral IL-17^+^/IFN-γ^+^ MAIT cells is significantly increased whereas this difference was not presented in CD4^+^T cells, suggesting that MAIT cells may preferably have Th17-biased function in COPD patients (**Figure 3F**).

### IL-17^+^ of MAIT Cells Correlated With Clinical Indicators in COPD Patients

**Figure 4:**
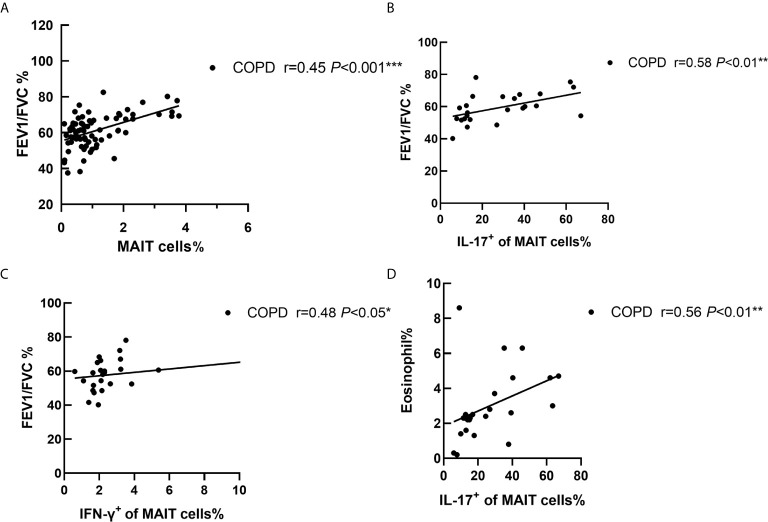
Correlation of cytokines of MAIT cells with clinical indicators in COPD patients. **(A)** The ratio of peripheral MAIT cells was positively correlated with FEV1/FVC (r = 0.45, *P* < 0.001). FEV1/FVC% was positive connected with IL-17^+^ of MAIT cells (r = 0.58, *P* < 0.01, **B**) and IFN-γ^+^ of MAIT cells (r = 0.48, *P* < 0.05, **C**). **(D)** Correlations analysis restricted to patients with COPD between proportion of eosinophils and IL-17+ of MAIT cells (r = 0.56, *P* < 0.01). Correlation coefficients were calculated using the Spearman rank method. *P < 0.05, **P < 0.01, ***P<0.001.

We then compared the correlation of IL-17^+^ MAIT cells with clinical variables. The results showed a positive correlation between MAIT cell frequency and post-bronchodilator FEV1/FVC (r = 0.45, *P* < 0.001; **Figure 4A**). Interestingly, percentage of IL-17^+^ MAIT cells were positively correlated with FEV1/FVC% (**Figure 4B**), denoted that IL-17^+^ MAIT cells play a potential role in COPD patients (r = 0.58, *P* < 0.01). We found the same condition in IFN-γ^+^ of MAIT cells (r = 0.48 P < 0.05, **Figure 4C**). As the GOLD grade increases, the proportion of IL-17^+^ MAIT cells gradually decreases (**Figure S3A**). Of note, in patients with symptom score B, frequency of IL-17^+^ MAIT cells was obviously added compared to score D (**Figure S3B**). IL-17^+^ of MAIT cells correlated with frequency of eosinophil in peripheral blood of COPD patients (r = 0.56, *P* < 0.01; **Figure 4D**). Finally. we evaluate correlation of smoke status, drug, and the frequency of hospitalization with IL-17^+^ MAIT cells. The evidence shows the proportion of patients with triple drug therapy and more than two hospitalizations have a lower production of IL-17 (**Figures S3C–E**).

### Increased Percentage of MAIT Cells in Lung in COPD Patients

**Figure 5:**
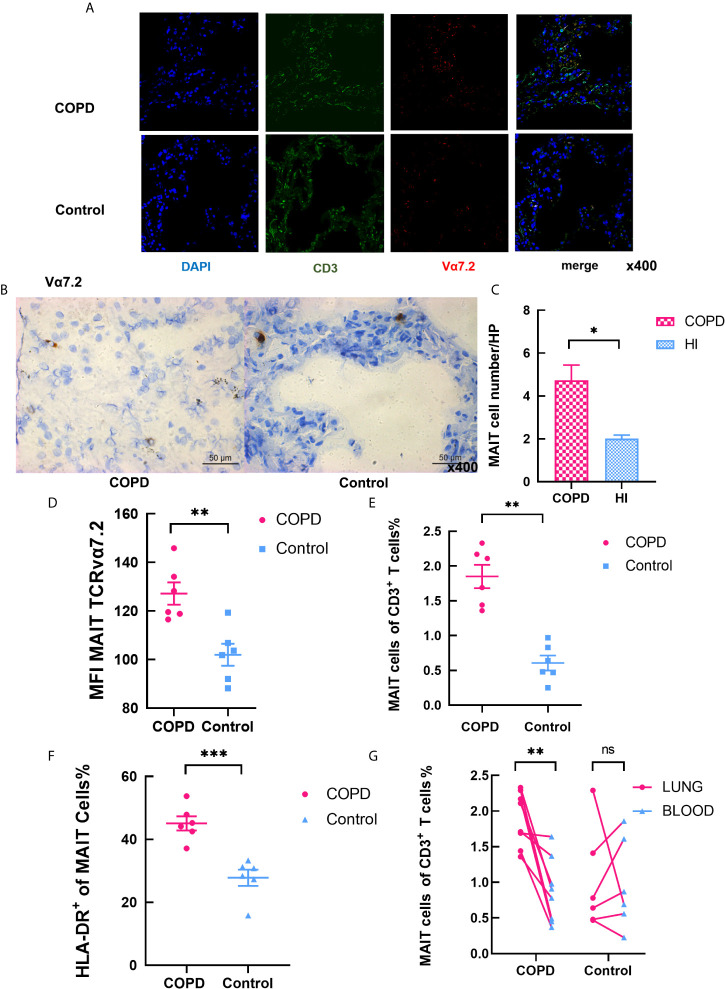
Increased levels of MAIT cells in the pulmonary parenchyma of COPD patients. **(A)** Representative immunofluorescence images for staining of CD3 (in green), TCRvα7.2 (in red), and DAPI (for nuclear in blue) in confocal laser. MAIT cells are pointed by the white arrows. **(B)** Immunohistochemical staining of Vα7.2 (original magnification, ×400). **(C)** The number of MAIT cells in COPD patients increased in high power lens. **(D)** MFI of Vα7.2 by semi-quantitative analysis under confocal condition. **(E)** Frequency of MAIT cells in lungs. **(F)** Elevated frequency of HLA-DR^+^ MAIT cells in COPD patients. **(G)** Comparison of MAIT cells in pulmonary parenchyma and peripheral blood in same individuals. Data were analyzed with Mann–Whitney U-tests. Horizontal lines are mean ± SEM values. *P < 0.05, **P < 0.01, ***P < 0.001.

Immunofluorescence staining for co-localization of MAIT cells defined by double-staining for Vα7.2 in combination with CD3, a phenotype identifying MAIT cells (23) (34). Clinical and laboratory characteristics of groups are summarized in **Table 2**. MAIT cells were accumulated around alveolar epithelial cells (**Figures 5A–B**). A fluorescent negative control was shown in **Figure S4**. The number of MAIT cells was increased with COPD patients (**Figure 5C**). The mean fluorescence intensity (MFI) of TCR vα7.2 measured by semi-quantitative analysis increased in COPD patients (mean 137.7 *vs.* 93.36, *P* < 0.01; **Figure 5D**). By analyzing the isolated primary lung cells through flow cytometry, there were significantly increasing of MAIT cells in COPD patients compared to controls (mean 1.85 *vs.* 0.61%, *P* < 0.01, **Figure 5E**) and HLA-DR marker is significantly activated (**Figure 5F**). Compared with lungs MAIT cells, the frequency of blood is greatly reduced in COPD (mean 1.85 *vs.* 0.88%, *P* < 0.01) and no difference in health group (mean 1.01 *vs.* 0.97; **Figure 5G**).

**Table 2:** Clinical and laboratory characteristics of lung tissue providers.

	Control Subjects	COPD Subjects
Total, n	6	6
Sex, Male/Female, n	6/0	6/0
Age (mean ± SD), years	68.33 ± 7.20	70.83 ± 3.44
BMI, kg/m^2^	23.10 ± 3.24	26.45 ± 1.86
**Clinical variables (mean ± SD)**		
FEV_1_ (ml)	2.35 ± 0.52	1.73 ± 0.43
FEV_1_ predicted (%)	103.52 ± 15.9	71.02 ± 16.90
FVC (ml)	2.58 ± 0.48	2.83 ± 0.60
FVC predicted (%)	91.26 ± 12.9	90.08 ± 16.39
FEV_1_/FVC (%)	89.66 ± 6.88	61.08 ± 8.97
FEF50 (%)	110.51 ± 24.93	37.28 ± 22.74
FEF25–75 (%)	104.42 ± 30.33	32.75 ± 12.24
Duration of COPD	NA	3.6 ± 1.92
GOLD stage,1/2/3/4	NA	2/3/1/0
**Laboratory variables (mean ± SD)**		
Leukocytes (cells/μl)	6.48 ± 1.08	6.96 ± 3.39
Neutrophils (cells/μl)	3.96 ± 0.78	4.531 ± 2.34
Lymphocytes (cells/μl)	1.82 ± 0.39	1.63 ± 0.61
Eosinophils (cells/μl)	0.22 ± 0.13	0.12 ± 0.09
Monocyte (cells/μl)	0.42 ± 0.07	0.63 ± 0.63
Hemoglobin (g/dl)	137.33 ± 11.74	137.83 ± 24.87
RDW (%)	13.38 ± 0.87	14.88 ± 3.7
Platelets (10^3^ cells/μl)	228 ± 79.93	264 ± 203.62
CRP level (mg/dl)	2.79 ± 2.70	3.5 ± 5.45
**Co-morbid conditions, n (%)**		
Hypertension	1 (16.7%)	3 (50%)
Diabetes mellitus	2 (33.3%)	1 (16.7)

**Figure 6:**
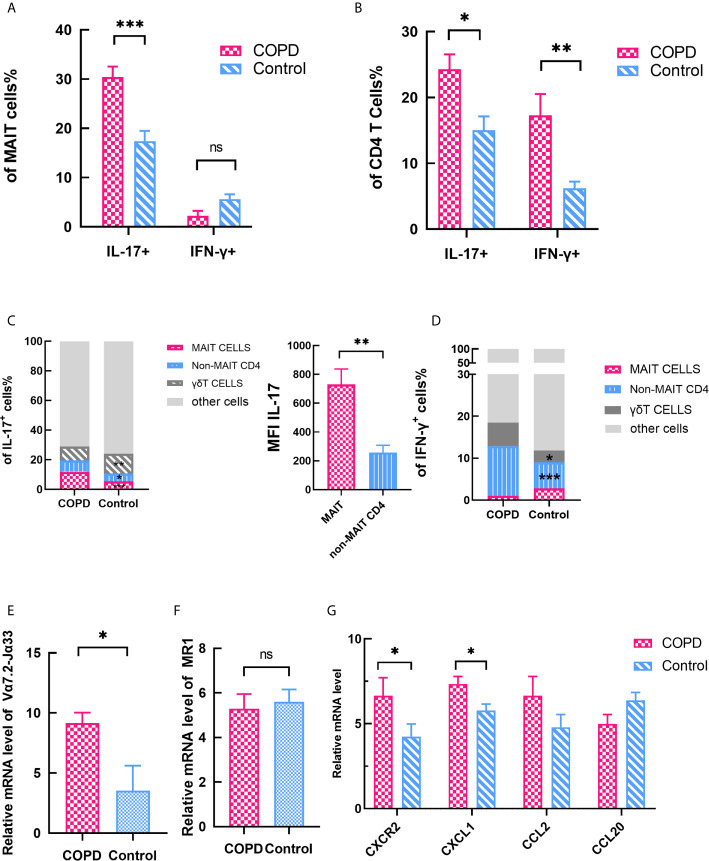
Expression of cytokines and chemokines in lung parenchyma. **(A)** The expression of IL-17^+^ and IFN-γ^+^ MAIT cells in lung. **(B)** The expression of IL-17^+^ and IFN-γ^+^ CD4 T cells in lung. **(C)** Data showing frequency of IL-17^+^ cells from COPD patients and control subset and showing mean fluorescent intensity (MFI) IL-17 of MAIT and non-MAIT CD4 cells. **(D)** Data showing frequency of IFN-γ^+^ cells from COPD patients and control subset. **(E)** Significantly elevated Vα7.2-Jα33 TCR gene expression was assessed in COPD patients. **(F)** MR1 gene expression did not differ in COPD patients and controls. **(G)** Expression levels of chemokine mRNAs in control and COPD patients. **(G)** Horizontal lines are mean ± SEM values. MR1, MHC-1 receptor. *P < 0.05, **P < 0.01, ***P < 0.001.

Moreover, we checked the cytokines secreted by of MAIT cells in lung. The frequency of IL-17^+^ of MAIT cells in lung significantly distinguished between patients and controls, and IFN-γ^+^ MAIT cells was no difference (**Figure 6A**). Notably, we found that IL-17^+^/IFN-γ^+^ non-MAIT CD4 T cells were significantly increased in both patients and controls (**Figure 6B**). Compared with control, the proportion of cells that produce IL-17 was remarkably increased including MAIT cells, γδT cells, non-MAIT cells in COPD patients, and IL-17 was expressed at significantly increased levels in MAIT cells as indicated by the mean fluorescent intensity (**Figure 6C**). Of note, the proportion of non-MAIT CD4 cells and γδT cells accounts for whole IFN-γ producing cells was increased in COPD patients while MAIT group is comparable (**Figure 6D**). It provides evidence that MAIT cells may play specific role in the lungs by shifting to the Th17 axis.

We also assessed the mRNA expression levels of pulmonary parenchyma. Vα7.2-Jα33 TCR mRNA level was significantly increased in COPD patients compared with control subjects (mean 9.15 *vs.* 3.53, *P* < 0.05, **Figure 6E**). Moreover, we checked the expression levels of the MAIT cell-engaged MR1 molecules. The result showed there is no difference in expression of MR1 molecules (mean 5.29 *vs.* 5.60, **Figure 6F**), indicating that MAIT cells are more likely to act in the lungs through cytokines-stimulate pathway. Previous report demonstrated CXCL1-CXCR2 axis (35) plays a crucial role in the recruitment of inflammatory cells in COPD. So we compared the chemokines between COPD patients and control groups. Chemokine mRNAs were found to be more strongly expressed in COPD tissues except CCL20 (**Figure 6G**), implying that MAIT cells are involved in the recruitment of what the periphery to the lung.

### IL-7 Enhances Type-17 Differentiation and Phenotype of MAIT Cells *In Vitro*

Previous studies have shown that MAIT cells are receptive to IL-18 (36) and IL-7 (37) stimulation to augment IL-17 production. We found MFI of IL-7R was remarkably decreased in COPD patients (mean 800.2 *vs.* 1742, *P* < 0.01, **Figure 8A**). Then we investigated the effects of IL-7 on MAIT cells. Exposed to IL-7 for 48 h, level of MFI CD127 was increased both in COPD and HI (**Figure 8B**). Expression of CCR5 was increased in COPD patients compared to HI (**Figure 8C**). After IL-7 was administered, we tested the expression of phosphorylated STAT3 at 30 min. The phosphorylation level was significantly increased in both COPD and health subsets (**Figure 8D**). At the same time, IL-7 upregulated type 17 signature molecules of RORγT expression at low levels *in vitro* (**Figure 8E**).

**Figure 7:**
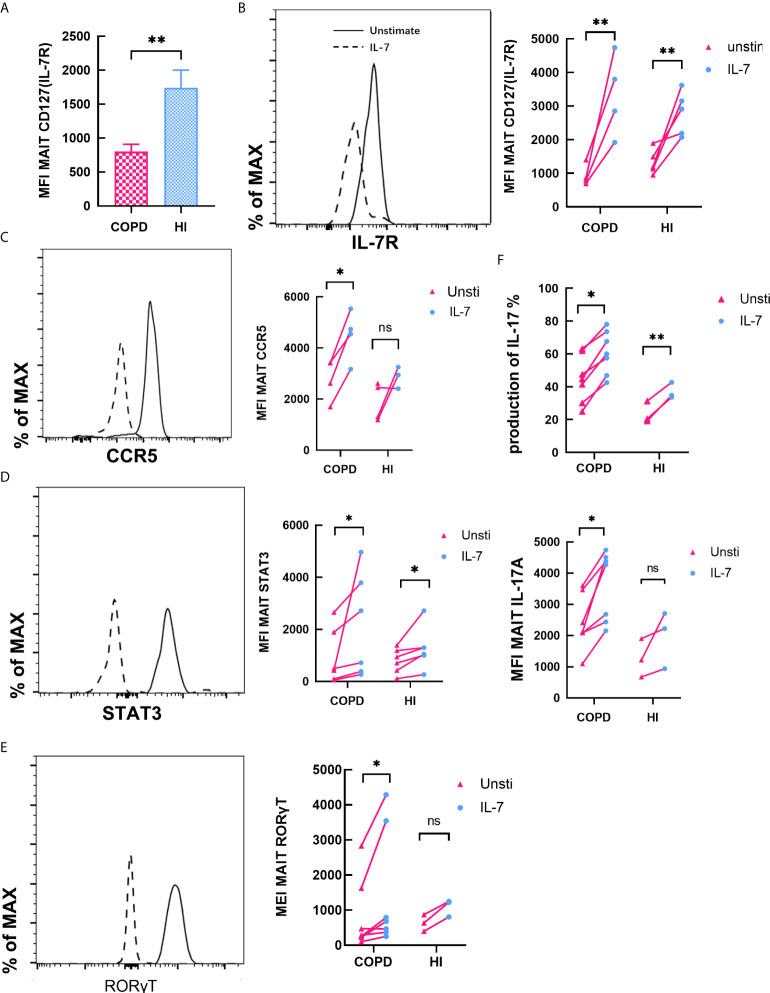
IL-7 enhances type-17 differentiation and phenotype of MAIT cells. **(A)** Expression of Il-7R in peripheral blood was downregulated in COPD patients. **(B, C)** CD127 and CCR5 expression by MAIT cells before and after treatment with IL-7 for 48 h in COPD and HI groups. **(D)** Quantification of phosphorylated STAT-3 in MAIT cells after treatment of PBMCs with IL-7 for 30 min both in COPD and health groups. **(E)** RORγT expression was quantified after treatment with IL-7 for 48 h. **(F)** Frequency and MFI of IL-17 among MAIT cells before and after treatment with IL-7. Horizontal lines are mean ± SEM values, *P < 0.05, **P < 0.01, ***P<0.001.

Finally, we compared the effect of PMA/ionomycin and IL-7 on the production of IL-17. It revealed an increase in the frequency of IL-17^+^ MAIT cells after IL-7 treatment in both patients and healthy individuals (mean 44.93 *vs.* 60.87%, *P* < 0.05; mean 23.70 *vs.* 37.03, *P* < 0.01, respectively). Mean fluorescence intensity (MFI) of IL-17 also confirmed this result (**Figure 7F**). All evidence demonstrated that IL-7 could significantly enhance type-17 differentiation on MAIT cells.

### γδT Cells Expressed Comparable Levels of IFN-γ but Higher Levels of IL-17

**Figure 8:**
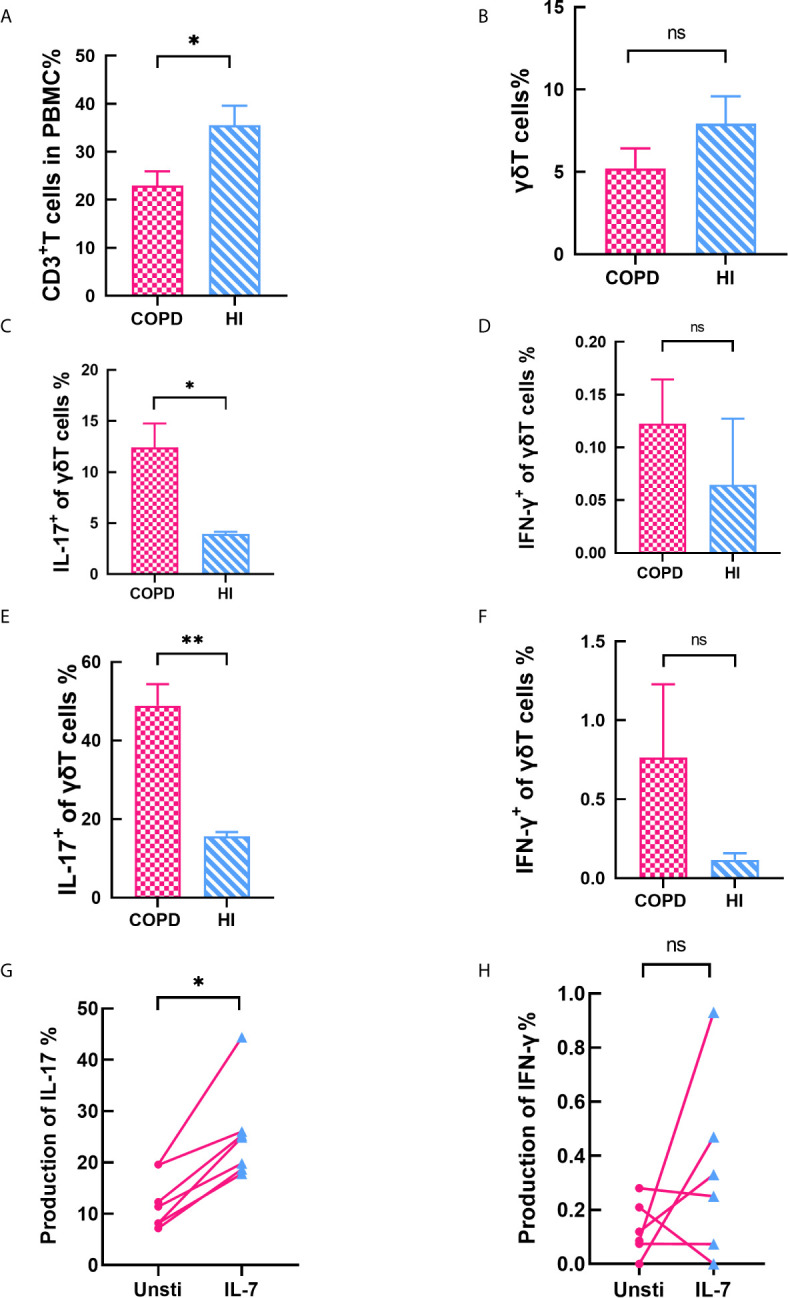
γδT cells expressed comparable levels of IFN‐γ but higher levels of IL‐17. **(A)** Frequency of CD3^+^T cells decreased in COPD patients. **(B)** Percentage of γδT cells was no difference between COPD and health. **(C–D)** IL-17^+^ γδT cells increased in peripheral blood while IFN-γ^+^ γδT cells makes no difference. **(E–F)** IL-17^+^ γδT cells increased in lung while IFN-γ^+^ γδT cells was equivalent. **(G–H)** IL-7 significantly stimulates the production of IL-17, but has no effect on IFN-γ. Horizontal lines are mean ± SEM values, *P < 0.05, **P < 0.01. ns, non-significant.

Finally, we performed a statistical analysis of another unconventional T cell. The total CD3^+^T cells in PBMC were found to decrease in COPD patients (mean 23 *vs.* 35.52%, p < 0.05, **Figure 7A**). Interestingly, percentage of γδT cells were no difference in COPD patients and health individuals (mean 5.21 *vs.* 7.93%, **Figure 7B**). We assessed cytokines production of γδT cells both in blood and pulmonary parenchyma. Our data indicated that γδT cells expressed comparable levels of IFN-γ but higher levels of IL-17 (**Figures 7C–F**). We also explored correlation with clinical parameters. Our data showed IL-17^+^ γδT cells did not change in the most severe patients, but ICS/LABA-used patients have reductive frequency (**Figure S5**). We found that IL-7 increases the production IL-17, but not IFN-γ (**Figures 8G–H**).

## Discussion

In this study, we assessed the phonotype and biological function of MAIT cells in COPD patients. Similar to the previous studies (25, 26), the amount of the MAIT cells significantly decreased in the COPD patients’ peripheral blood, but their activities were highly stimulated. Circulating MAIT cells migrated to lung parenchyma, produced high level of Th17 type cytokines and low Th1 type cytokines, therefore exerted potential ability against local inflammation.

Similar with results observed in metabolic diseases (23, 34, 38), our data revealed that circulating MAIT cell deficiency was accompanied by upregulation of HLA-DR, and CCR5 expression, indicating that reduction in MAIT cell numbers in peripheral blood of COPD patients might be due partly to the migration of MAIT cells to inflamed tissues. Nevertheless, the “migration theory” was not supported in bronchial biopsy and bronchoalveolar lavage fluid (26) in COPD patients. And the proportion of MR1-tetramer^+^ MAIT cells were lower in Mtb-infected bronchial epithelium in children (39). It is however notable that the subset of MAIT cell consistent sequences was enriched in lung granuloma tissues compared to Mtb-infected individuals (40). MAIT cells were significantly higher in BALF from patients with parenchymal infiltration (41) and children with pneumonia (42). The discrepancy may be attributed to some factors. For example, MR1-5-OP-RU Tetramer (15), IL-18R and PLZF co-expression (43), and TRAV1-2+ semi-invariant TCRα (40) could all be identified as MAIT cells except the enriched expression of CD161. It has been hypothesized that reduced tetramer binding affinity and increased variability underestimated the production of MAIT cells in bronchoalveolar lavage fluid (26). These need to be further explored.

Due to the high expression of its activation signal CD69, PD-1 (22), and 7-AAD (44), we proposed a new hypothesis that the reduction of MAIT cells was due to activation-induced cell death. Our results also found that glucocorticoid use significantly reduced the proportion of MAIT cells in peripheral blood. Previous data indicated that glucocorticoids inhibit T cell proliferation, and MAIT cells have been shown to be inhibited in SLE (44), asthma (45), and multiple sclerosis (46) after treatment with prednisone. In addition, frequency of peripheral blood CD3^+^T cells, iNKT cells (25), and γδ T cells (11) were found to be significantly reduced in COPD patients. Our data showed only a downward trend in γδ T cells in patients, but there was no statistic difference from the control group. These findings suggest that the decline of MAIT cells count in peripheral blood may be a common phenomenon of T cells due to activation depletion of cells rather than a phenomenon specific to MAIT cells.

Research on sources of IL-17 in COPD has previously focused mainly on CD4^+^ Th17 cells with controversial results (47). It has been documented that IL-17 can induce CXCL8 and CXCL5, participating in the recruitment of neutrophils (6). IL-17 also affects most of the cells in the parenchyma, such as macrophages and DCs, expressing IL-17 receptors and synthesizing pro-inflammatory factors such as IL-6 and TNF-α (48). Several lines of clinical studies have established that elevated levels of IL-17 associated with both stable and acute exacerbations of COPD (47). However, different IL-17 producing unconventional T cell is relevant to protective effects of inflammation. γδT cells can accelerate the resolution of pulmonary inflammation (49). Changes in lung function caused by inflammation and emphysema are strongly dependent on iNKT cells (50). We concluded for the first time that IL-17 increased produced by MAIT cells and concluded IL-17^+^ MAIT cells play an important role in COPD patients. Interestingly, we found that the proportion of IL-17 was positively correlated with peripheral eosinophils. Recent evidence indicates that eosinophil change may predict clinical response to ICS therapy (51) and high eosinophil values were statistically inferred for acute exacerbations (52). However, it has also been reported that eosinophils have no diagnostic value for COPD (53). The diagnostic efficacy and ROC curve analysis suggested that the threshold value of percentage of MAIT cells for identifying hospitalization for severe acute exacerbations be 1.3% (**Figure S6**). The value showed a sensitivity of 85.1% and a specificity of 52.6%. Taking the value as a critical point, the area under the curve (AUC) was 0.69 and the standard error of mean was 0.059 (*P* < 0.01). Our results demonstrate that MAIT cells can be used as a co-marker with eosinophils to predict admission to acute exacerbations of COPD.

Though MAIT differed from Th17 cells in the differentiated genes, the key transcriptional mechanisms that govern IL-17 expression were shared between MAIT and Th17 cells. In the present study, IL-17 was expressed at significantly increased levels of MAIT cells by level of MFI. The high expression of RORγt and STAT3 in human MAIT cells, indicated the polarization of IL-17. MAIT cells produced more IFN-γ in pleural effusion accumulation (54). In contrast, breast ductal epithelial cells (55), oral epithelium (43) showed Th17-skewed response in the face of bacterial infection. Notably, Th2 bias response only was found in NAFLD patients (23).Therefore, the phenotype diversity of MAIT cells *in vitro* needs further study.

Our results showed that there was no difference in the expression of MR1 in the lung tissues between COPD and control groups. This indicates that cytokines preferentially stimulate activation of MAIT cells rather than TCR stimulation *in vivo*. CD127 expression was associated with cell sensitive apoptosis and IL-7 is a potential candidate for rescuing T-cell apoptosis in COPD (56). Our preliminary experiment indicated MFI of IL-7R was decreased in COPD patients. There is a remarkable increase of IL-7 in COPD patients in serum. In lung parenchyma, IL-7 expression has increased trend in patients with COPD, although there was no statistic difference (**Figure S7**). Our data revealed treatment with IL-7 resulted in increased production of IL-17, upregulation of IL-7R and related transcription factors. This may indicate the functional relationship between IL-7 signaling pathway and MAIT-17 differentiation *in vivo*, which may be due not only to increased activation, but more likely to greater differentiation of MAIT cells into Th17 in COPD patients.

Our study had several limitations. First, healthy female participants were more than those with COPD. Although we observed changes occur independent of gender. No significant difference between men and women was found in Chinese individuals with healthy circulating frequency MAIT cells (57). Similarly, sex discrepancy of MAIT cells was only found in reproductive female (58), while we chose the elderly female. On the other hand, we conducted a comparison of healthy individuals of sex group. We found no difference in peripheral blood MAIT cell frequencies between health individuals who were male or female (*P* = 0.786) so we assumed that there was no difference between men and women in the control group, and for patients with COPD, our current conclusion was that there also was no difference (*P* = 0.888). Another limitation was that the bronchial mucosal MAIT cells need to be further identified, although there is a higher percentage of cells from the lung parenchyma, which can be investigated by alveolar lavage or immunostaining of the airway. Because the pulmonary data were exploratory in nature, findings will need further replication in a larger cohort.

In summary, we assessed the phenotype and function of MAIT cells in COPD patients. Our comparative analysis between peripheral blood and pulmonary parenchyma of COPD patients raises the possibility that activated MAIT cells may migrate into the inflamed tissue. Of importance, the levels of MAIT cells appear predictive of the course of the COPD. It is noteworthy that IL-17A secretion correlates with the positive evolution of clinical parameters, raising the possibility that MAIT cells play a potential role, which also applied to γδT cells. Elucidating the mechanisms of the depletion and activation of the unconventional T cells may be key to the development of IL-17 targeted therapy in COPD. The joint diagnosis of MAIT cells needs to be validated in large-scale clinical trials.

## Data Availability Statement

The original contributions presented in the study are included in the article/**Supplementary Material**. Further inquiries can be directed to the corresponding authors.

## Ethics Statement

The studies involving human participants were reviewed and approved by the ethics committee of Huadong Hospital. The patients/participants provided their written informed consent to participate in this study.

## Author Contributions

WQ, HG, and HZ: designed experiments. WQ, NK, YW, YC, and LX recruited study volunteers and collected essential human samples. WQ, NK, and YC conducted experiments and analyzed experimental data. WQ, HG, and HZ wrote the manuscript. All authors contributed to the article and approved the submitted version.

## Funding

This study was supported by National Natural Science Foundation of China (81871100,81600056), Shanghai Natural Science Foundation (18ZR1412900), Bethune Research and Development Fund Project (BJ-RW2020002J), and Guanghua Medical Fund Project.

## Conflict of Interest

The authors declare that the research was conducted in the absence of any commercial or financial relationships that could be construed as a potential conflict of interest.
