# Supplementary material for: Mucosal Associated Invariant T Cells Were Activated and Polarized Toward Th17 in Chronic Obstructive Pulmonary Disease
Source: Front Immunol. 2021 Mar 31;12:640455. doi: 10.3389/fimmu.2021.640455 (PMC8044354; doi:10.3389/fimmu.2021.640455)
Supplement: Supplementary file 1 [file DataSheet_1.pdf]

Supplement

Figure1

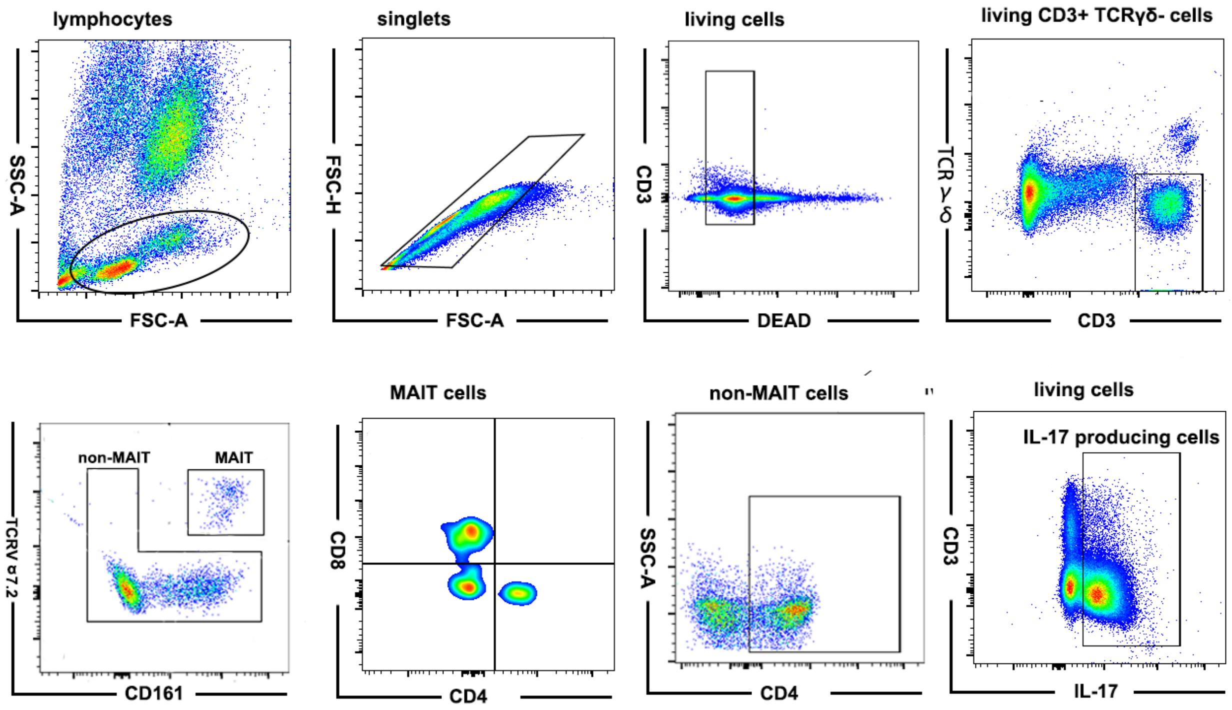

Representative gating strategy for identification of MAIT cells ($CD3^{+}TCR\gamma\delta^{-}$ $CD161^{high}$ $V\alpha7.2^{+}$) and non-MAIT $CD4^{+}$T cells excluding dead cells.

Figure2

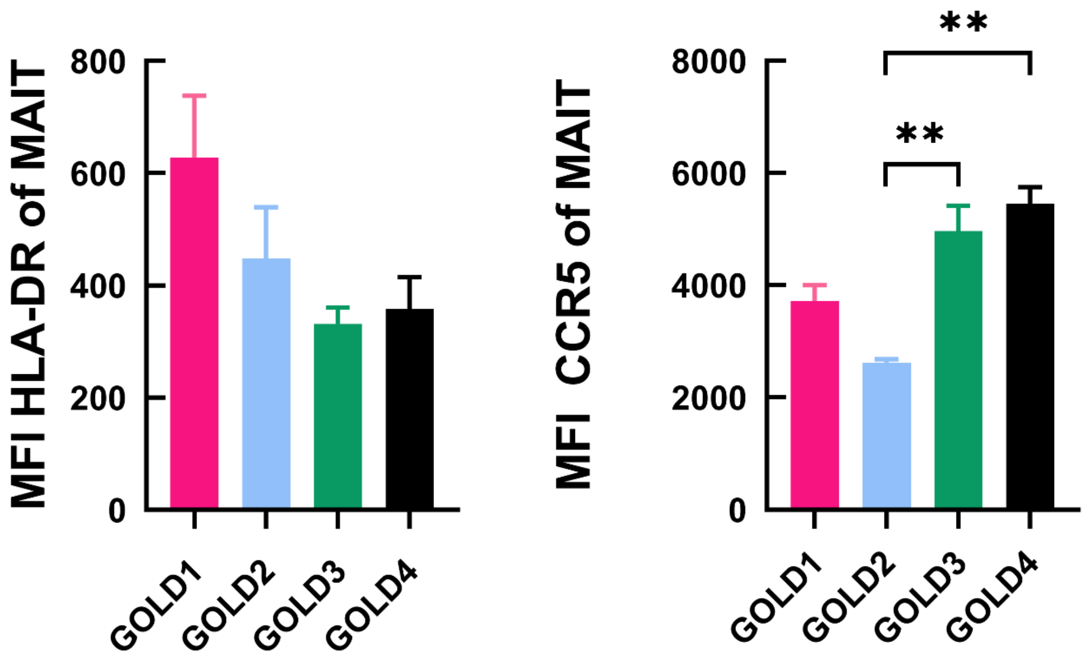

Correlation between MFI of HLA-DR, CCR5 with COPD severity. The MFI of HLA-DR was decreased, though not statistic significant (*P*=0.08). The MFI of CCR5 was increased in GOLD3 and GOLD4 patients compared to GOLD2 (mean 2626 vs. 4962,5447, *P*<0.01).

Figure3

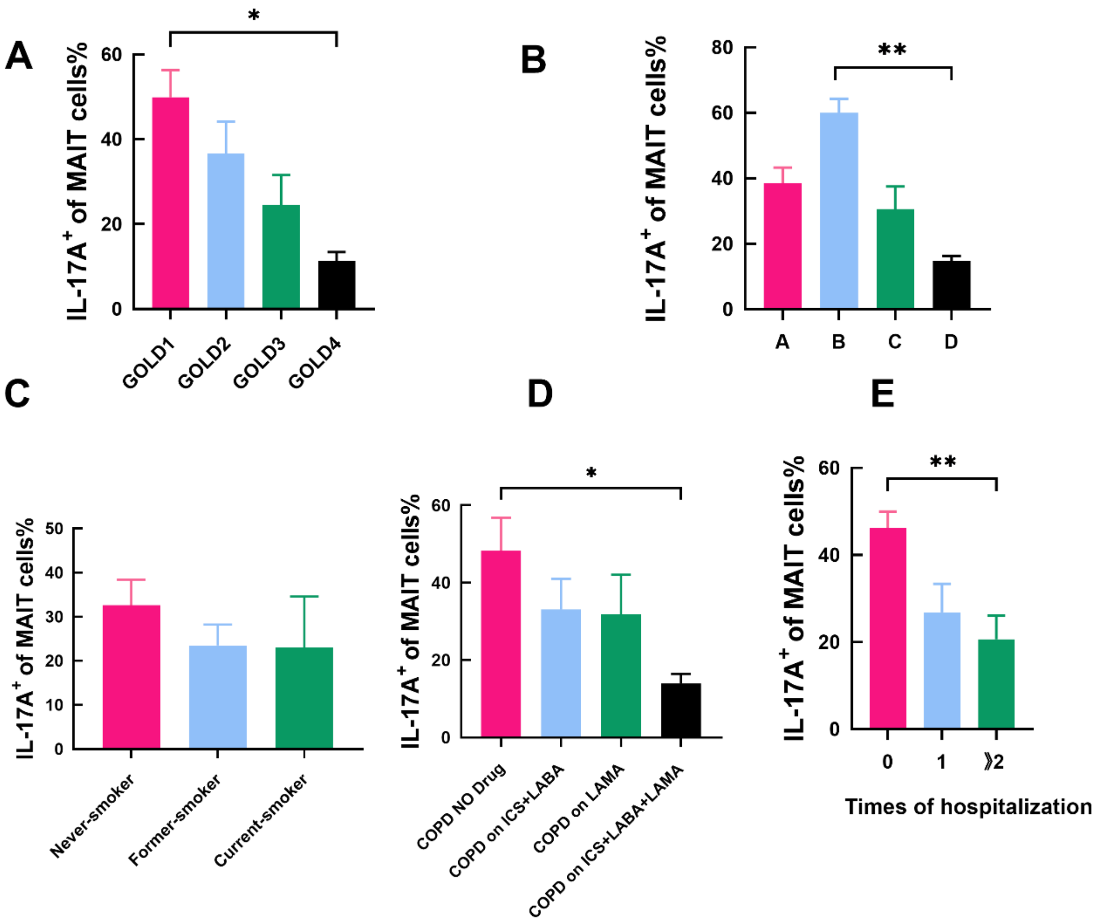

Correlation of IL-17⁺ MAIT cells with clinical indicators. (A) The proportion of IL-17⁺ MAIT cells gradually decreases as the GOLD grade increases. (B)The proportion of IL-17⁺ MAIT cells in symptom assessment. Correlation of the frequencies of IL-17⁺ cells among MAIT cells with smoke status (C), drug (D) and times of hospitalization (E). Horizontal bars represent the mean±SEM values. Statistics: Kruskal-Wallis test, *P < 0.05; **P < 0.01; ***P< 0.001

Figure4

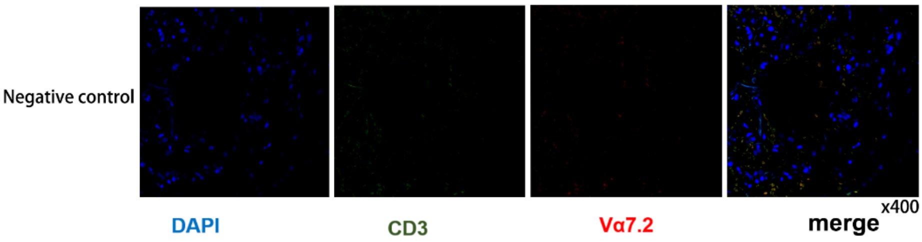

Representative immunofluorescence images for staining of CD3 (in green), TCRvα7.2 (in red) and DAPI (for nuclear in blue) in confocal laser.

Figure5

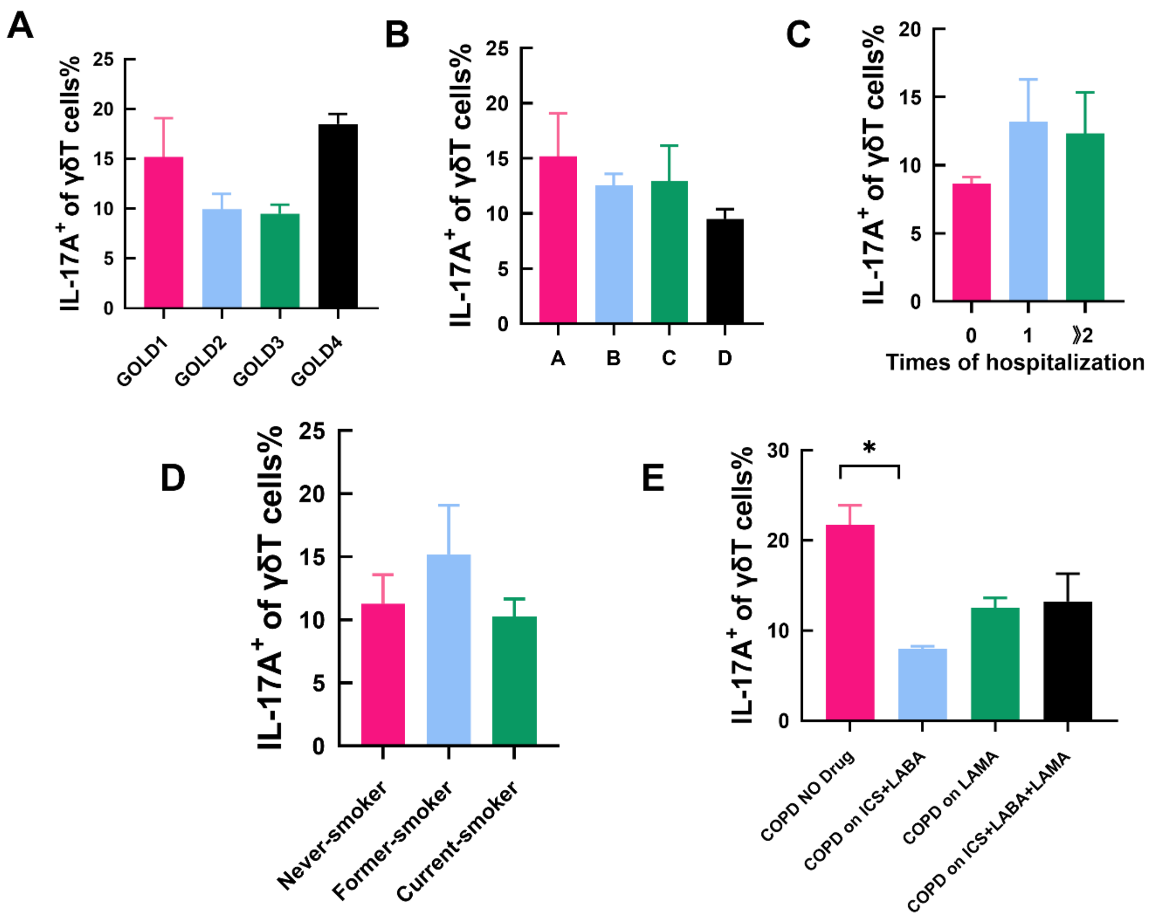

Correlation of IL-17⁺ γδT cells with clinical indicators. (A) Correlation between percentage of IL-17⁺ γδT cells with COPD grades. (B) Correlation between percentage of IL-17⁺ γδT cells with symptom assessment. (C) Correlation between percentage of IL-17⁺ γδT cells with times of hospitalization. (D) Proportion of IL-17⁺ γδT cells in peripheral blood according to smoking status. (E) Proportion of IL-17⁺ γδT cells in peripheral blood according to drug-using. Horizontal bars represent the mean±SEM values. Statistics: Kruskal-Wallis test, *P < 0.05.

Figure6

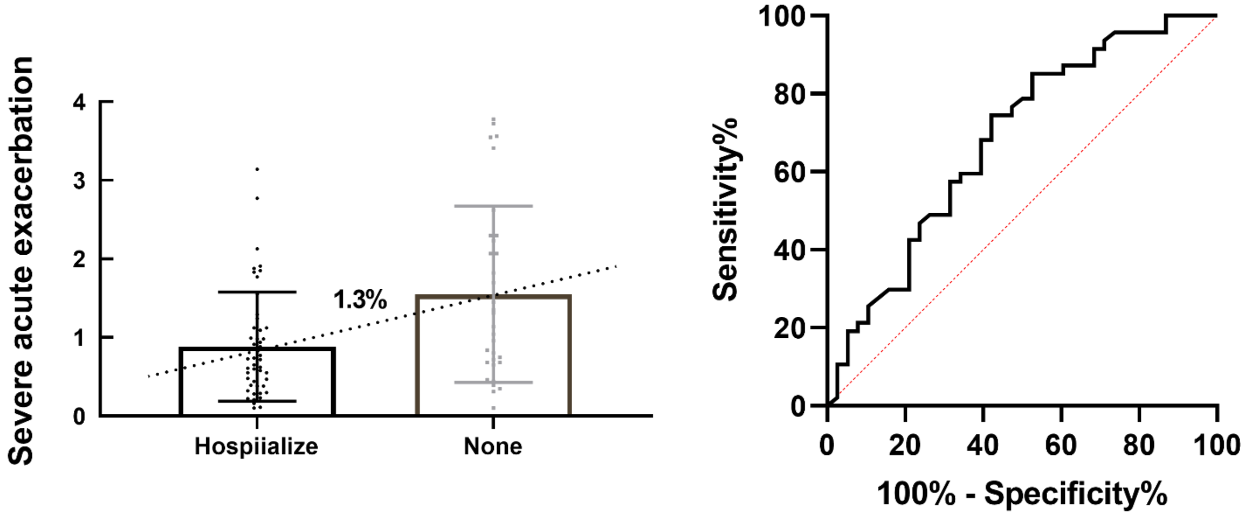

ROC curve analysis suggested that the threshold value of percentage of MAIT cells for identifying hospitalization for severe acute exacerbations be 1.3%. The value showed a sensitivity of 85.1% and a specificity of 52.6%.

Figure7

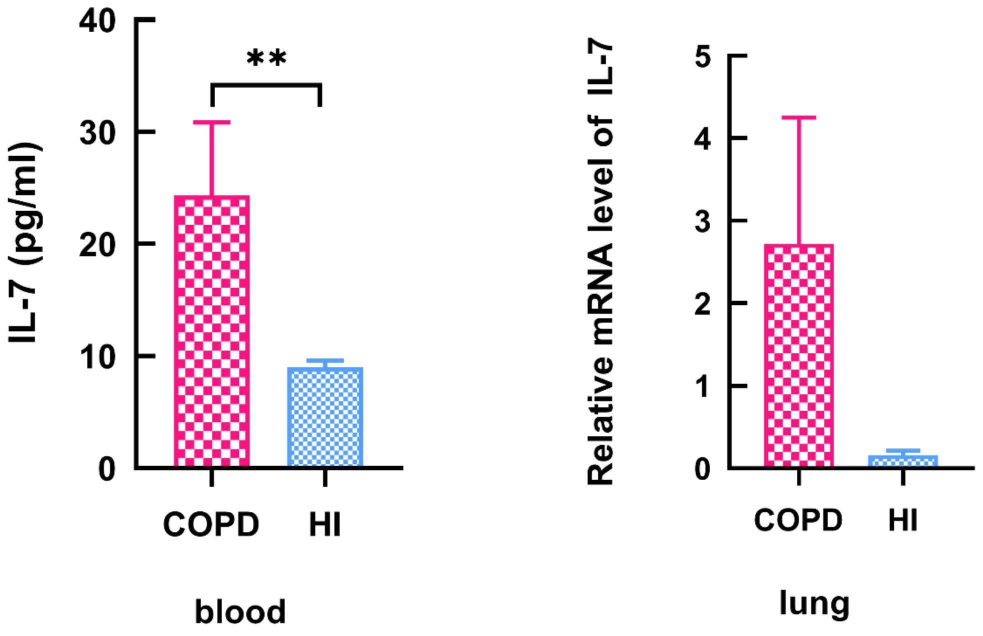

There is a remarkable increased of IL-7 in COPD patients in serum. In lung parenchyma, IL-7 expression has increased trend in patients with COPD, although there was no statistic difference. Horizontal bars represent the mean±SEM values. Statistics: Mann-Whitney test, ***P* < 0.01.
